# Optimization of protease production from *Rhizomucor miehei* Rm4 isolate under solid-state fermentation

**DOI:** 10.1186/s43141-022-00358-9

**Published:** 2022-05-30

**Authors:** Houthail Alahmad Aljammas, Sabah Yazji, Abdulhakim Azizieh

**Affiliations:** Department of Food Sciences, Faculty of Agricultural Engineering, Damascus University, Damascus, Syria

**Keywords:** Optimization, Protease, *Rhizomucor miehei*, Response surface methodology, One-factor-at-a-time

## Abstract

**Background:**

Protease is one of the most important industrial enzymes. The importance of protease bioproduction comes from meeting the increasing demand for this enzyme especially in the cheese industry. *Rhizomucor miehei* protease is the preferred substitute for the traditional rennet. Solid-state fermentation (SSF) shows promising results in enzyme production. An optimization strategy was applied to optimize the production of *Rhizomucor miehei* protease in a solid medium. The components of the fermentation medium were screened by using the one-factor-at-a-time (OFAT) approach. The optimization process then was performed by using the response surface methodology (RSM) approach based on five factors (fermentation time, temperature, pH, moisture content, nitrogen concentration) at five levels. Specific milk clotting activity and milk clotting activity/proteolytic activity ratio were considered as response variables in the optimization process.

**Results:**

Among several combinations, wheat bran was selected as the best substrate. Casein was selected based on preliminary screening of nitrogen sources. The optimal conditions identified by RSM analysis were found to be 81.21 h, 41.11°C, 6.31, 80%, and 1.33% for fermentation time, temperature, pH, moisture content, and casein concentration, respectively. The performed fermentation process under the optimized conditions gave an enzymatic extract with the values of 5.11 mg/mL, 2258.13 Soxhlet unit/mL, 441.90 Soxhlet unit/mg, 1.14 protease unit/mg, and 388.66 for protein content, milk clotting activity, specific clotting activity, specific proteolytic activity, and milk clotting activity/proteolytic activity ratio, respectively. The aforementioned values were close to the predicted values.

**Conclusion:**

The high milk clotting activity and the relatively low proteolytic activity signify higher specificity of the produced enzyme, which is favorable in cheese making. The observed results reveal the efficiency of the applied statistical approaches in obtaining desired values of response variables and minimizing experimental runs, as well as achieving good predictions for response variables.

## Background

Proteases are considered as one of the most important groups of industrial enzymes, representing about 60% of the global market share [43]. Besides detergent [21], food industries are the main application of proteases, where they are used in baking [28], meat tenderization [14], synthesis of aspartame [15], preparation of protein hydrolysates [32], and dairy manufacturing [45]. The primary function of protease, in cheese making, is to initiate the milk coagulation process, through rapid and highly specific cleavage of the major milk proteins (casein). Due to the worldwide shortage of the calf rennet, many efforts have been made to search for alternative milk coagulants [45]. Mucorpepsin (EC 3. 4. 23. 23) produced by *Rhizomucor miehei* has been proven to be a sufficient substitute for the traditional rennet, due to its high specificity in splitting the Phe105-Met106 bond of κ-casein similar to chymosin performance. It also shows high milk clotting activity and relatively low proteolytic activity; similar sensitivity to temperature, pH, and calcium ions to that of calf rennet; good cheese quality; and lower incidence of bitter flavor [19, 38].

SSF offers several advantages over liquid fermentation, as reducing both capital and operating expense, simple downstream processes, and lower in cost. Relatively cheap materials such as agro-industrial residues can be used as substrates in SSF. In addition, the concentrated nature of the solid substrate and the low moisture content reduce the contamination and increase the volumetric productivity [8]. Furthermore, SSF is particularly suitable for fungal enzyme production [23].

The OFAT approach involves changing one parameter at a time while others are being fixed until the optimal conditions are achieved. Despite the simple implementation of this procedure, it does not take into account the interactions between variables. OFAT also can be time-consuming and requires more runs. On the other hand, RSM enables the evaluation of the interactions among the studied factors and minimizes the number of experimental runs [4, 49].

Several studies have recently used the OFAT approach combined with RSM for the optimization of enzyme production [4, 35, 42]. This strategy could help in the preliminary screening of the candidate variables which will be chosen in the subsequent design with a fewer number of the experimental runs [35].

In the present study, we aimed to maximize the production of *Rhizomucor miehei* protease through optimization of SSF conditions by OFAT and RSM approaches.

## Methods

### Fungal isolate

*Rhizomucor miehei* Rm4 was selected among several isolates that were previously isolated from the Syrian soil based on the results of previous research [7], where isolates were screened based on the yield and activity of the produced protease. It was maintained on PDA slants and stored at 4 °C for further use.

### Inoculum preparation

Molds were inoculated onto 90-mm Petri dishes containing 20 mL of PDA and incubated at 37 °C for 5 days. The inoculum was obtained by scraping the PDA surface in the presence of 30 mL of sterilized distilled water under sterile conditions. The concentration of spore suspension was determined by counting on a Neubauer counting chamber.

### Preparation of fermentation media

Wheat bran, wheat flour, soybean, and corn were obtained from the local markets. Corn and soybean were ground and then sieved with a metal sieve (1 mm). A mineral salt solution was prepared of the composition (g/L) ZnSO_4_ · 7H_2_O: 0.07, MgSO_4_· 7H_2_O: 0.07, CuSO_4_· 7H_2_O: 0.07, and FeSO_4_: 0.09. Ten milliliters of this solution was diluted to 1 L with distilled water [46]. Substrates were moisturized with a specific volume of the diluted nutrient solution according to the statistical design. Twenty grams of the moist substrates were distributed in each of 250-mL Erlenmeyer flasks, and flasks were plugged with cotton and autoclaved for 20 min at 121 °C. After cooling, media were inoculated with spore suspension (10^6^ spores/mL) with the ratio of 10% under sterile conditions. The pH was adjusted initially by adding a specific volume of 0.1 N HCl or 0.1 N NaOH (within the desired total moisture content) until the desired pH is reached.

### Experimental design and statistical analysis

#### Preliminary screening of medium formulation

The components of the fermentation medium were selected by the OFAT approach, based on the amount and activity of the produced enzyme in each formulation. Wheat bran was used as a base substrate, and it was partially replaced with wheat flour, corn powder, and soybean powder separately at the ratios of 10:90, 20:80, 30:70, 40:60 (substitute:wheat bran). After substrate selection, the effect of nitrogen source was studied, where casein, yeast extract, and peptone were supplemented to the substrate at the ratios of 1, 2, 3, 4, and 5% w/w. The most effective source was selected near the most positive significant levels of addition. The cultivation was carried out at 37°C and moisture content of 60% for 6 and 5 days for substrates and nitrogen source selection steps, respectively.

Preliminary screening experiments were performed in triplicate, and results were expressed as the mean ± standard deviation. The results were analyzed by the analysis of variance test (ANOVA) and the least significant difference (LSD) at the significant level of 0.01 using IBM SPSS Statics 21.

#### Optimization of protease production by RSM

A central composite design (CCD) was applied to optimize the variable levels and minimize the number of experiments, where five factors (fermentation time, temperature, pH, moisture content, casein concentration) were selected at five levels: −*α* (minimum), −1 (low), 0 (central), +1 (high), and +*α* (maximum) with equaled distance from the central points (0) to each of factorial points (±1) and axial points (±*α*), respectively, for each factor (Table 1). CCD was derived from the combination of different points (±*α*, 0, ±1) of the studied factors using Minitab 17 statistical software, where 47 experimental runs were generated (Table 2). The results of CCD were statistically analyzed by Minitab 17 statistical software. The multiple regression analysis was used to generate second-order polynomial equations which describe the effect of independent factors on the response variables. Statistical analysis of the resulted models was carried out by ANOVA test. The fit of the regression models was assessed by the coefficient of determination (*R*^2^) and the significance of the models at the significance levels (*P*-value) of 0.01 and 0.05.

**Table 1:** Coded levels of independent variables employed in the CCD

Independent variables	Symbol	Unit	Intervals and levels				
			−***α***	−1	0	+1	+***α***
Fermentation time	X1	Hour	24	48	72	96	120
Temperature	X2	°C	30	35	40	45	50
pH	X3	-	3	4	5	6	7
Moisture content	X4	% v/w	40	50	60	70	80
Casein concentration	X5	% w/w	0.5	1	1.5	2	2.5

**Table 2:** The experimental runs of CCD for optimization of protease production

Run order	Factor					Blocks
	X1	X2	X3	X4	X5	
1	48	35	4	50	1.0	1
2	96	35	4	50	2.0	1
3	48	45	4	50	2.0	1
4	96	45	4	50	1.0	1
5	48	35	6	50	2.0	1
6	96	35	6	50	1.0	1
7	48	45	6	50	1.0	1
8	96	45	6	50	2.0	1
9	48	35	4	70	2.0	1
10	96	35	4	70	1.0	1
11	48	45	4	70	1.0	1
12	96	45	4	70	2.0	1
13	48	35	6	70	1.0	1
14	96	35	6	70	2.0	1
15	48	45	6	70	2.0	1
16	96	45	6	70	1.0	1
17	48	35	4	50	2.0	1
18	96	35	4	50	1.0	1
19	48	45	4	50	1.0	1
20	96	45	4	50	2.0	1
21	48	35	6	50	1.0	1
22	96	35	6	50	2.0	1
23	48	45	6	50	2.0	1
24	96	45	6	50	1.0	1
25	48	35	4	70	1.0	1
26	96	35	4	70	2.0	1
27	48	45	4	70	2.0	1
28	96	45	4	70	1.0	1
29	48	35	6	70	2.0	1
30	96	35	6	70	1.0	1
31	48	45	6	70	1.0	1
32	96	45	6	70	2.0	1
33	72	40	5	60	1.5	1
34	72	40	5	60	1.5	1
35	72	40	5	60	1.5	1
36	72	40	5	60	1.5	1
37	72	40	5	60	1.5	1
38	24	40	5	60	1.5	2
39	120	40	5	60	1.5	2
40	72	30	5	60	1.5	2
41	72	50	5	60	1.5	2
42	72	40	3	60	1.5	2
43	72	40	7	60	1.5	2
44	72	40	5	40	1.5	2
45	72	40	5	80	1.5	2
46	72	40	5	60	0.5	2
47	72	40	5	60	2.5	2

#### Enzyme extraction

After the incubation period, 100 mL of distilled water (4°C) was added to the solid fermentation medium. The flasks were shaken at 220 rpm for 1 h. The extract was filtered through Whatman paper (No. 1), and the obtained filtrate was centrifuged at 5000 rpm for 20 min at 4°C. The supernatant was used as a crude enzyme [24].

### Enzyme assay

#### Protein content

The protein content of the crude extract was determined according to the method of Lowry et al. [33], and bovine serum albumin (BSA) was used to generate the standard curve.

#### Milk clotting activity (MCA)

MCA was determined according to the method of Arima et al. [6] and expressed in terms of Soxhlet units (SU). One SU is defined as the quantity of enzyme required to clot 1 mL of the substrate containing 0.1 g skimmed milk powder and 0.0014 g calcium chloride in 40 min at 35°C and was calculated with the formula:

$$\mathrm{unit}\ \mathrm{of}\ \mathrm{milk}-\mathrm{clotting}\ \mathrm{activity}\ \left(\mathrm{U}\right)=\frac{2400}{\mathrm{T}}\times \frac{\mathrm{S}}{\mathrm{E}}\times \frac{35}{\mathrm{t}}$$

where *T* is the time required for clotting, *S* is the milk volume, *E* is the enzyme volume, and *t* is the temperature of the reaction.

#### Proteolytic activity (PA)

PA was determined by a modified method of Kunitz [30]. One milliliter of the crude enzyme was added to 1 mL of 1% casein in 0.1 M Sorensen’s phosphate buffer (pH 6), and the mixture was incubated at 35°C for 20 min. The reaction then was terminated by the addition of 3 mL of 5% trichloroacetic acid solution. The mixtures were left for 1 h at 25°C and then were centrifuged at 5000 rpm for 10 min. After filtration, the content of liberated amino acids and peptides in the supernatant was determined according to Lowry et al. [33] at 750 nm. Tyrosin was used to generate the standard curve. One unit of protease activity (PU) was defined as the activity which liberates 1 μg of tyrosin per minute, under the measurement conditions.

## Results

### Preliminary screening of medium formulation

Wheat bran medium with no replacement gave the most positive significant response variables, followed by corn powder, wheat flour, and soybean powder supported media, respectively. A gradual decrease in response values was observed by increasing replacement ratios (Table 3). According to the results, wheat bran was chosen as the sole substrate without replacement.

**Table 3:** The effect of medium formulation on protease production by *Rhizomucor miehei*

Medium formulation	Protein content (mg/mL)	Milk clotting activity (SU/mL)	Specific activity (SU/mg)	Proteolytic activity (PU/mL)	MCA/PA
Wheat bran	1.44 ± 0.04^a^	364.6 ± 30.94^a^	253.19 ± 14.33^a^	1.2 ± 0.06^a^	301.81 ± 9.30^a^
Wheat bran replaced with corn powder					
10%	1.36 ± 0.04^b^	314.66 ± 9.24^b^	231.36 ± 0.05^b^	1.44 ± 0.13^ab^	220.03 ± 12.63^b^
20%	1.32 ± 0.01^b^	296.44 ± 11.4^bc^	213.15 ± 6.77^b^	1.38 ± 0.10^ac^	216 ± 7.41^b^
30%	1.16 ± 0.01^c^	275.8 ± 5.72^cde^	237.75 ± 3.14^ab^	1.61 ± 0.17^bcd^	172.19 ± 13.37^c^
40%	1.14 ± 0.01^cd^	265.09 ± 6.03^dh^	232.27 ± 3.86^b^	1.21 ± 0.10^a^	219.99 ± 14.02^b^
Wheat bran replaced with wheat flour					
10%	1.44 ± 0.03^a^	295.33 ± 11.48^be^	205.09 ± 3.63^c^	2.08 ± 0.25^be^	142.16 ± 10.72^d^
20%	1.18 ± 0.01^c^	221.23 ± 12.71^fg^	187.09 ± 8.87^e^	1.28 ± 0.05^ad^	172.92 ± 6.45^c^
30%	1.1 ± 0.01^de^	206.89 ± 3.57^gi^	188.12 ± 2.44^e^	2.64 ± 0.20^f^	78.64 ± 5.01^ef^
40%	1.08 ± 0.01^e^	205.13 ± 14.55^gi^	189.52 ± 11.88^ce^	1.5 ± 0.17^ad^	136.84 ± 7.52^d^
Wheat bran replaced with soybean powder					
10%	1.52 ± 0.02^f^	237.95 ± 9.9^hf^	156.54 ± 4.93^f^	2.47 ± 0.22^f^	96.24 ± 4.83^f^
20%	1.58 ± 0.01^g^	198.2 ± 7.31^gi^	125.44 ± 4^g^	2.72 ± 0.24^f^	72.99 ± 3.82^e^
30%	1.78 ± 0.004^h^	192 ± 6.78^ij^	108.18 ± 3.57^h^	2.71 ± 0.10^f^	71.01 ± 0.19^e^
40%	1.52 ± 0.003^f^	169.04 ± 7.37^j^	111.21 ± 4.65^gh^	1.9 ± 0.08^de^	88.94 ± 0.94^ef^

The results are expressed as mean ± SD. Within columns, values followed by the same letter(s) are not significantly different at the 0.01 level

### The effect of nitrogen sources

Casein was found to be the best nitrogen source for protease production followed by peptone (3%) and yeast extract (1%), respectively. The maximum MCA and specific milk clotting activity (spMCA) were obtained by addition of casein at the ratio of 2%, whereas the maximum MCA/PA ratio was obtained by addition of casein at the ratio of 1%. The negative effect was significantly appeared in all response variables with higher concentrations of nitrogen sources (Table 4). According to the results, casein was selected near the most significant levels (1 and 2%) for further optimization.

**Table 4:** The effect of nitrogen source on protease production by *Rhizomucor miehei*

Type and concentration of nitrogen source	Protein content (mg/mL)	Milk clotting activity (SU/mL)	Specific activity (SU/mg)	Proteolytic activity (PU/mL)	MCA/PA
Blank	2.11 ± 0.01^ab^	444 ± 19.50^a^	210.43 ± 7.96^a^	0.92 ± 0.06^a^	481.7 ± 11.27^a^
Yeast extract					
1%	2.11 ± 0.02^a^	555.42 ± 23.43^b^	263.23 ± 8.65^b^	0.94 ± 0.06^a^	591.45 ± 14.46^b^
2%	2.19 ± 0.04^bcd^	424.14 ± 15.61^a^	193.67 ± 4.03^ac^	0.94 ± 0.06^a^	450.42 ± 19.29^ac^
3%	2.13 ± 0.04^ab^	370.28 ± 9.39^c^	173.84 ± 1.88^c^	1.33 ± 0.07^b^	279.48 ± 8.03^d^
4%	2.13 ± 0.05^ab^	324.32 ± 22.60^d^	152.26 ± 7.82^d^	1.38 ± 0.08^b^	243.92 ± 5.81^e^
5%	2.10 ± 0.04^a^	231.75 ± 20.41^e^	110.36 ± 7.83^e^	1.41 ± 0.10^b^	160.89 ± 7.40^f^
Peptone					
1%	2.11 ± 0.03^ab^	571.42 ± 19.51^b^	270.82 ± 7.09^bf^	1.46 ± 0.10^b^	392.04 ± 15.20^g^
2%	2.13 ± 0.03^ab^	648.64 ± 27.44^fg^	304.53 ± 8.61^gh^	2.34 ± 0.10^dc^	278.75 ± 14.31^d^
3%	2.14 ± 0.04^ab^	666.66 ± 24.57^f^	311.52 ± 6.72^g^	2.46 ± 0.20^d^	272.23 ± 24.91^d^
4%	2.12 ± 0.04^ab^	613.89 ± 20.42^g^	289.57 ± 4.87^fhkm^	1.92 ± 0.30^e^	320.51 ± 15.35^h^
5%	2.08±0.04^a^	571.42 ± 18.35^b^	274.72 ± 3.90^bk^	1.30 ± 0.16^b^	438.79 ± 9.88^c^
Casein					
1%	2.23±0.04^c^	827.58 ± 20.25^h^	371.11 ± 3.59^n^	1.40 ± 0.07^b^	591.46 ± 23.51^b^
2%	2.27±0.06^c^	857.14 ± 20.81^h^	377.59 ± 1.91^n^	1.62 ± 0.09^be^	527.76 ± 20.62^i^
3%	2.38±0.05^e^	666.66 ± 23.61^f^	280.11 ± 4.63^g^	2.02 ± 0.10^ce^	328.87 ± 11.84^h^
4%	2.24±0.03^cd^	666.66 ± 19.11^f^	297.62 ± 4.25^g^	5.26 ± 0.27^f^	127.27 ± 3.96^j^
5%	2.15±0.03^ad^	648.64 ± 19.42^fg^	301.69 ± 4.95^gm^	5.98 ± 0.32^g^	108.62 ± 2.49^j^

The results are expressed as mean ± SD. Within columns, values followed by the same letter(s) are not significantly different at the 0.01 level

### Optimization of protease production by RSM

The experimental results of the RSM design are shown in Table 5. Yield and enzyme quality are supposed to be chosen as response variables to optimize the production process, where higher values of protein content and MCA, as well as lower PA, are preferred. The highest values of MCA, protein content, spMCA, and MCA/PA ratio were obtained at Run 47, Run 43, Run 43, and Run 29, respectively, whereas the lowest values of both PA and specific proteolytic activity (spPA) were obtained at Run 17. Among all performed experiments, Run 6, Run 7, Run 16, Run 43, Run 45, and Run 47 showed the most desirable values for all response variables. However, the multi-response optimization method was applied to evaluate the optimal experimental conditions for protease production and obtain a composite desirability of all the response variables. spMCA and MCA/PA ratio were selected for multi-response analysis as expressive variables which describe other response variables where higher spMCA is favorable with respect to higher values of MCA/PA.

**Table 5:** Observed values for the response variables

Run order	Milk clotting activity (SU/mL)	Proteolytic activity (PU/mL)	Enzymatic content (mg/mL)	Specific MCA (SU/mg)	Specific PA (PU/mg)	MCA/PA
1	40.00	0.62	0.67	59.61	0.92	64.52
2	66.67	0.92	2.11	31.60	0.44	72.46
3	41.18	0.78	2.01	20.49	0.39	52.79
4	54.42	0.82	2.22	24.46	0.37	66.36
5	475.11	1.92	2.34	203.32	0.82	247.45
6	1333.33	5.18	3.58	372.44	1.45	257.45
7	1372.55	5.26	4.01	342.50	1.31	261.15
8	936.45	5.69	3.31	282.92	1.72	164.65
9	15.38	0.79	1.85	8.30	0.43	19.49
10	405.80	2.19	3.05	133.05	0.72	185.20
11	545.17	2.77	3.35	162.74	0.83	196.67
12	868.70	4.03	3.55	244.55	1.13	215.83
13	666.67	4.09	3.47	192.11	1.18	163.18
14	1014.49	4.57	3.96	256.01	1.15	221.82
15	800.00	5.19	3.18	251.64	1.63	154.22
16	1372.55	4.92	3.67	374.40	1.34	279.02
17	20.00	0.15	1.30	15.34	0.12	133.33
18	44.79	0.32	1.45	30.84	0.22	139.98
19	205.88	1.30	1.68	122.21	0.77	158.37
20	535.51	2.82	3.10	172.55	0.91	189.56
21	307.69	2.36	2.82	109.29	0.84	130.24
22	1296.30	5.56	4.42	292.99	1.26	233.04
23	1372.55	4.79	3.99	344.00	1.20	286.45
24	857.14	5.55	3.87	221.48	1.43	154.44
25	190.22	1.30	2.06	92.48	0.63	145.80
26	331.91	2.27	2.40	138.39	0.95	146.08
27	750.00	4.18	4.23	177.47	0.99	179.30
28	441.18	2.15	3.06	144.10	0.70	205.06
29	486.11	1.23	3.39	143.56	0.36	396.83
30	750.00	2.17	3.83	195.76	0.57	344.83
31	897.44	2.96	3.62	248.02	0.82	303.13
32	1043.48	4.29	3.79	275.32	1.13	243.26
33	800.00	2.57	3.79	211.13	0.68	311.00
34	827.59	3.90	2.81	294.25	1.39	212.43
35	923.08	3.35	3.37	273.96	1.00	275.20
36	1122.99	5.37	3.46	324.93	1.55	209.23
37	1090.91	3.99	3.81	286.42	1.05	273.23
38	0	0.35	0.71	0.00	0.50	0.00
39	297.12	1.35	3.12	95.31	0.43	219.61
40	11.76	0.22	1.27	9.25	0.17	53.48
41	393.44	1.84	3.55	110.70	0.52	214.35
42	28.57	0.19	0.90	31.75	0.21	150.38
43	1453.29	6.95	3.54	410.84	1.96	209.15
44	990.61	6.24	3.14	315.68	1.99	158.87
45	1521.74	4.70	3.39	448.28	1.99	323.88
46	257.35	2.52	2.27	113.37	1.11	102.04
47	1544.12	4.85	4.24	363.96	1.14	318.18

The CCD analysis resulted in two second-order polynomial equations (models 1 and 2) for the prediction of response variables in terms of coded factors as follows:

1 $$\mathrm{spMCA}=-4180+3{\mathrm{X}}_5-27.4\ {\mathrm{X}}_4+349\ {\mathrm{X}}_3+169.3\ {\mathrm{X}}_2+15.94\ {\mathrm{X}}_1-31.7\ {{\mathrm{X}}_5}^2+0.279\ {\mathrm{X}}_4-12.3\ {{\mathrm{X}}_3}^2-2.104\ {{\mathrm{X}}_2}^2-0.0966\ {{\mathrm{X}}_1}^2-0.80\ {\mathrm{X}}_5\ast {\mathrm{X}}_4-2.8\ {\mathrm{X}}_5\ast {\mathrm{X}}_3+2.81\ {\mathrm{X}}_5\ast {\mathrm{X}}_2+0.944\ {\mathrm{X}}_5\ast {\mathrm{X}}_1-2.68\ {\mathrm{X}}_4\ast {\mathrm{X}}_3+0.190\ {\mathrm{X}}_4\ast {\mathrm{X}}_2+0.0355\ {\mathrm{X}}_4\ast {\mathrm{X}}_1+0.10\ {\mathrm{X}}_3\ast {\mathrm{X}}_2+0.299\ {\mathrm{X}}_3\ast {\mathrm{X}}_1-0.1449\ {\mathrm{X}}_2\ast {\mathrm{X}}_1.$$

2 $$MCA/PA =-3457+283\ {\mathrm{X}}_5-2\ {\mathrm{X}}_4+314\ {\mathrm{X}}_3+101.7\ {\mathrm{X}}_2+12.23\ {\mathrm{X}}_1-19.6\ {{\mathrm{X}}_5}^2+0.029\ {{\mathrm{X}}_4}^2-12.5\ {{\mathrm{X}}_3}^2-0.958\ {{\mathrm{X}}_2}^2-0.0520\ {{\mathrm{X}}_1}^2-2.46\ {\mathrm{X}}_5\ast {\mathrm{X}}_4+13.0\ {\mathrm{X}}_5\ast {\mathrm{X}}_3-2.22\ {\mathrm{X}}_5\ast {\mathrm{X}}_2-0.5\ {\mathrm{X}}_5\ast {\mathrm{X}}_1-0.14\ {\mathrm{X}}_4\ast {\mathrm{X}}_3+0.061\ {\mathrm{X}}_4\ast {\mathrm{X}}_2+0.0441\ {\mathrm{X}}_4\ast {\mathrm{X}}_1-3.16\ {\mathrm{X}}_3\ast {\mathrm{X}}_2-0.409\ {\mathrm{X}}_3\ast {\mathrm{X}}_1-0.097\ {\mathrm{X}}_2\ast {\mathrm{X}}_1.$$

ANOVA results for spMCA and MCA/PA models are shown in Tables 6 and 7, respectively, where the significance of main effects and interactions for all factors are described.

**Table 6:** ANOVA for the spMCA model

Source	DF	Adj SS	Adj MS	*F*-value	*P*-value
Model	21	595702	28367	6.94	0.000
Blocks	1	477	477	0.12	0.736
Linear	5	142855	28571	6.99	0.000
Casein	1	1305	1305	0.32	0.577
Moist	1	230	230	0.06	0.814
pH	1	15532	15532	3.80	0.063
Temp	1	24552	24552	6.01	0.022
Time	1	101236	101236	24.77	0.000
Square	5	198436	39687	9.71	0.000
Casein*casein	1	1829	1829	0.45	0.510
Moist*moist	1	22750	22750	5.57	0.026
pH*pH	1	4387	4387	1.07	0.310
Temp*temp	1	80744	80744	19.76	0.000
Time*time	1	90479	90479	22.14	0.000
2-way interaction	10	45021	4502	1.10	0.310
Casein*moist	1	510	510	0.12	0.727
Casein*pH	1	65	65	0.02	0.901
Casein*temp	1	1584	1584	0.39	0.539
Casein*time	1	4109	4109	1.01	0.326
Moist*pH	1	22905	22905	5.60	0.026
Moist*temp	1	2876	2876	0.70	0.409
Moist*time	1	2325	2325	0.57	0.458
pH*temp	1	8	8	0.00	0.965
pH*time	1	968	968	0.24	0.631
Temp*time	1	9673	9673	2.37	0.136
Error	25	102166	4087		
Lack-of-fit	21	95141	4531	2.58	0.185
Pure error	4	7025	1756		
Total	46	697867

**Table 7:** ANOVA for the MCA/PA model

Source	DF	Adj SS	Adj MS	*F*-value	*P*-value
Model	21	206978	9856.1	1.63	0.120
Blocks	1	5507	5506.9	0.91	0.349
Linear	5	59713	11942.6	1.98	0.117
Casein	1	2318	2317.8	0.38	0.541
Moist	1	22	22.2	0.00	0.952
pH	1	18782	18781.7	3.11	0.090
Temp	1	7748	7748.0	1.28	0.268
Time	1	30844	30843.6	5.11	0.033
Square	5	43398	8679.6	1.44	0.246
Casein*casein	1	698	698.0	0.12	0.737
Moist*moist	1	250	250.0	0.04	0.840
pH*pH	1	4545	4544.9	0.75	0.394
Temp*temp	1	16732	16732.3	2.77	0.108
Time*time	1	26218	26218.1	4.34	0.048
2-way interaction	10	27716	2771.6	0.46	0.901
Casein*moist	1	4834	4833.8	0.80	0.379
Casein*pH	1	1344	1344.0	0.22	0.641
Casein*temp	1	984	984.3	0.16	0.690
Casein*time	1	1157	1157.1	0.19	0.665
Moist*pH	1	62	62.2	0.01	0.920
Moist*temp	1	300	300.0	0.05	0.825
Moist*time	1	3588	3588.0	0.59	0.448
pH*temp	1	7988	7988.4	1.32	0.261
pH*time	1	3089	3088.8	0.51	0.481
temp*time	1	4369	4368.9	0.72	0.403
Error	25	150943	6037.7		
Lack-of-fit	21	143167	6817.5	3.51	0.116
Pure error	4	7776	1943.9		
Total	46	357921			

The spMCA model was significant at the significance level of 0.01 (*p* ˂ 0.01). Linear and quadratic effects of the time factor on spMCA were significant at the significance level of 0.01. Linear and quadratic effects of temperature on spMCA were significant at the significance levels of 0.05 and 0.01, respectively. The quadratic effect of moisture was insignificant at the significance level of 0.01 but significant at the 0.05 level, whereas the linear effect was not significant at 0.01 or 0.05 levels. For pH and casein, neither the linear nor the quadratic effect was significant on spMCA. The interaction effect between pH and moisture was significant at 0.05 level. The interactions between other factors had no significant effect at 0.01 or 0.05 levels.

The overall regression model for MCA/PA was not significant at 0.01 or 0.05 levels. The effects of all independent factors were not significant on MCA/PA ratio, except for the effect of the time factor which was significant at the level of 0.05.

The coefficients of determination (*R*^2^) obtained for spMCA and MCA/PA models were 85.36% and 57.83%, respectively.

Insignificant terms were eliminated from the spMCA model in order to reduce the model by removing one term each time and repeating the regression, until only significant terms remain, with respect to their hierarchical structure, where non-significant linear terms were included in the final reduced model if quadratic or interaction terms of these variables were found to be significant [16]. The significance criterion of 0.05 was used to eliminate insignificant terms from the model. The final reduced model was expressed as follows:

$$\mathrm{spMCA}=-4332-11\ {\mathrm{X}}_5-24.9\ {\mathrm{X}}_4+349\ {\mathrm{X}}_3+169.8\ {\mathrm{X}}_2+18.08\ {\mathrm{X}}_1-31.7\ {{\mathrm{X}}_5}^2+0.279\ {{\mathrm{X}}_4}^2-12.3\ {{\mathrm{X}}_3}^2-2.104\ {{\mathrm{X}}_2}^2-0.0966\ {{\mathrm{X}}_1}^2-0.80\ {\mathrm{X}}_5\ast {\mathrm{X}}_4+2.81\ {\mathrm{X}}_5\ast {\mathrm{X}}_2+0.944\ {\mathrm{X}}_5\ast {\mathrm{X}}_1-2.68\ {\mathrm{X}}_4\ast {\mathrm{X}}_3+0.190\ {\mathrm{X}}_3\ast {\mathrm{X}}_2+0.229\ {\mathrm{X}}_3\ast {\mathrm{X}}_1-0.1449\ {\mathrm{X}}_2\ast {\mathrm{X}}_1$$

ANOVA results for the final reduced model for spMCA are shown in Table 8. *R*^2^ value obtained for the final reduced model of spMCA was 85.02.

**Table 8:** ANOVA for the reduced model of spMCA

Source	DF	Adj SS	Adj MS	*F*-value	*P*-value
Model	18	593305	32961	8.83	0.000
Blocks	1	477	477	0.13	0.724
Linear	5	153419	30684	8.22	0.000
Casein	1	1305	1305	0.35	0.559
Moist	1	10794	10794	2.89	0.100
pH	1	15532	15532	4.16	0.051
Temp	1	24552	24552	6.57	0.016
Time	1	101236	101236	27.11	0.000
Square	5	198436	39687	10.63	0.000
Casein*casein	1	1829	1829	0.49	0.490
Moist*moist	1	22750	22750	6.09	0.020
pH*pH	1	4387	4387	1.17	0.288
Temp*temp	1	80744	80744	21.62	0.000
Time*time	1	90479	90479	24.23	0.000
2-way interaction	7	42624	6089	1.63	0.168
Casein*moist	1	510	510	0.14	0.714
Casein*temp	1	1584	1584	0.42	0.520
Casein*time	1	4109	4109	1.10	0.303
Moist*pH	1	22905	22905	6.13	0.020
Moist*temp	1	2876	2876	0.77	0.388
pH*Time	1	968	968	0.26	0.615
Temp*time	1	9673	9673	2.59	0.119
Error	28	104563	3734	-	-
Lack-of-fit	24	97538	4064	2.31	0.216
Pure error	4	7025	1756	-	-
Total	46	697867	-	-	-

**Fig. 1:**
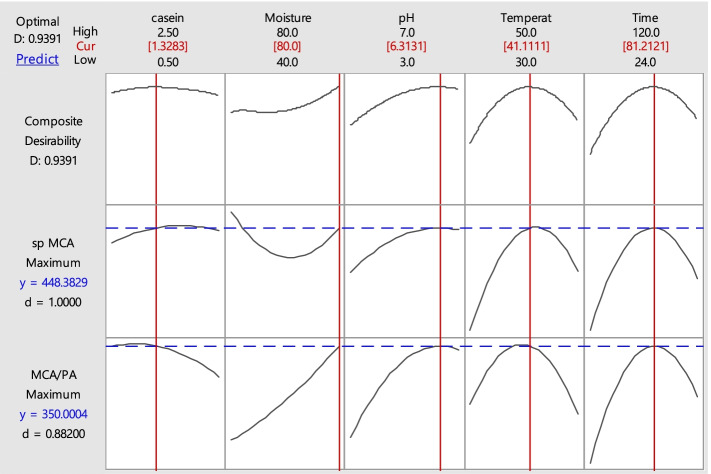
Multiple response optimization of protease production. Columns represent the effect of each factor on the responses (rows). Vertical red lines represent the optimal values of the independent variables yielding the desired responses. Horizontal blue lines represent the predicted values of responses for the optimal set of independent variables. The numbers in the first row represent the range for each factor and its optimal value (shown in red). Optimal values of responses are shown in the first column (blue values). *D* is the composite desirability index, encompassing all responses; *d* is the individual desirability index. (0≤di≤1) higher index values reveal higher desirability, whereas 1 represents a completely desirable

**Fig. 2:**
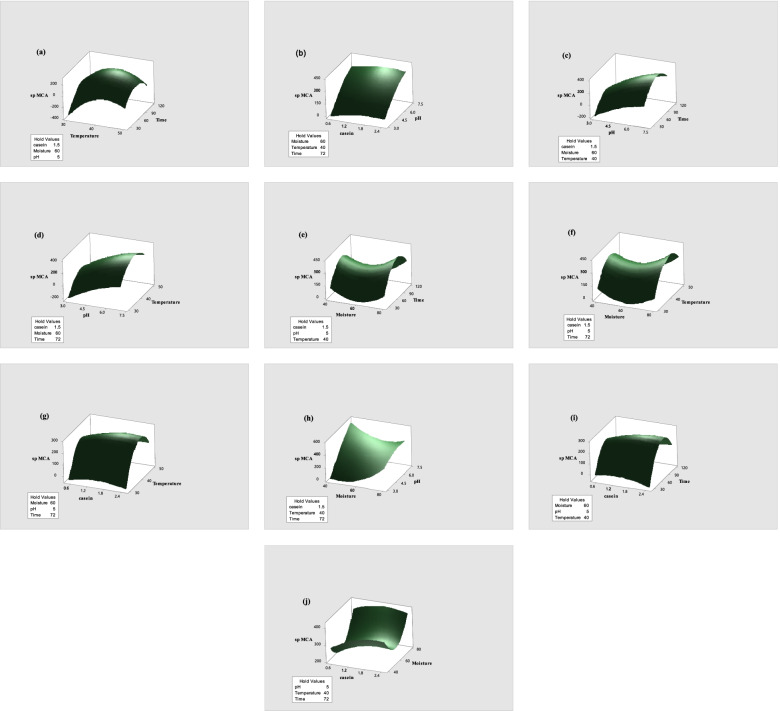
3D Response surface plots representing the interaction between each two independent variables and their effect on spMCA. Other variables were kept at their constant level. **a** spMCA vs temperature; time, **b** spMCA vs casein; pH, **c** spMCA vs pH; time, **d** spMCA vs pH; temperature, **e** spMCA vs moisture; time, **f** spMCA vs moisture; temperature, **g** spMCA vs casein; temperature, **h** spMCA vs Moisture; pH, **i** spMCA vs casein; time, **j** spMCA vs casein; moisture

Figure 1 shows the optimal values of the independent variables and their effects on response variables. Under the optimized conditions, a production process was established to validate the multiple response prediction. The obtained values of protein content, MCA, spMCA, PA, spPA, and MCA/PA ratio were 5.11 mg/mL, 2258.13 SU/mL, 441.90 SU/mg, 5.81 PU/mL, 1.14 PU/mg, and 388.66, respectively; these values were close to the predicted values based on the composite desirability approach. Figure 2 represents the interaction between each two independent variables and their effect on spMCA of protease while maintaining other variables at the central point.

## Discussion

### Preliminary screening of medium formulation

The variation of enzyme production in the different media is due to the differences in the physical and chemical properties among the substrates.

The size and geometric shape of substrate particles are the most important physical characteristics which affect medium porosity and determine the surface-area-to-volume ratio which affects the rate of reactions [8]. The fine wheat flour particles may get packed together and filled the space between wheat bran particles, which affected the microbial respiration and, consequently, the growth rate.

As for the chemical properties that appear to affect protease production, they could be represented by the structure and availability of substrates, the type and proportions of organic materials, and the nutrient content.

The decrease in protease production with an increasing replacement ratio with soybean powder is due to the decreasing carbon-to-nitrogen (C/N) ratio. The C/N ratios in the used substrates are approximately 39.1:1.26, 44.7:2.20, 50.70:2.28, 45.40:6.71 for wheat flour, wheat bran, corn, and soybean, respectively [17, 44]. A reduction in protease production from filamentous fungi with decreasing C/N ratio was indicated by Aguilar et al. [3] and Boratyński et al. [13].

High concentrations of easy assimilation saccharides have a negative effect on protease production despite stimulating growth [31, 34]. The high amount of reducing sugars resulted from the hydrolysis of starch in wheat flour and corn powder may have led to carbon catabolite repression, suggesting that in the absence of glucose, protease plays a role in supplying peptides or amino acids as the carbon or energy source in addition to being a nitrogen source. Consequently, protease synthesis could be repressed when the energy status of the cells is high in the presence of glucose [40]. This phenomenon is widespread in filamentous fungi [2]. Amer et al. [9] have reported inhibition of protease production from *Rhizomucor miehei* with increasing glucose concentration in the fermentation medium. They also observed that higher lactose concentration resulted in lower enzyme activity and high biomass production, and they suggested that the glucose that resulted from lactose breakdown has led to protease repression or a metabolic deviation for biomass synthesis. In contrast to our findings, Thakur et al. [46] obtained higher protease production from *Rhizomucor miehei* by the supplementation with wheat flour to wheat bran medium.

The reduced enzymatic activity observed in media containing soybean and corn powder may be due to the presence of protease inhibitors in these substrates [1, 27]. Similar findings related to the reduction of protease production by the supplementation with corn flour and wheat flour to wheat bran medium were reported by Khademi et al. [29].

The higher activity obtained by the wheat bran medium is due to the moderate C/N ratio and the sufficient nutrient needed by microorganisms for growth and production of enzymes as well as the high surface area/volume ratio. Foda et al. [26] indicated that the wheat bran medium gave the highest MCA of *Rhizomucor miehei* protease among several agro-industrial residues.

Organic nitrogen sources have been reported to be favorable for protease production [11, 20, 34, 36]. Casein, yeast extract, and peptone had a positive effect on protease production at the optimal range, among them casein was the superior, contrary to the finding of Khademi et al. [29] that the supplementation with 1% peptone to wheat bran has a negative effect on protease production from *Rhizomucor miehei*; they also argued that yeast extract is superior to casein in protease production enhancement.

As reported earlier, the enhancement of MCA of *Rhizomucor miehei* protease can be obtained by supplementation with skim milk powder [26, 37, 46] and casein [26, 41]. The higher MCA obtained by supplementation with casein is due to the stimulation of enzyme synthesis by the targeted substrate.

The reduction of protease production observed at the higher nitrogen source concentrations is due to nitrogen metabolite repression. According to this mechanism, the genes encoding enzymes involved in the utilization of metabolically “costly” nitrogen sources are not expressed when favorable nitrogen sources are available [12, 48]. Similar findings have been reported [26, 29, 41].

### Optimization of protease production by RSM

The occurrence of the desired values of MCA, PA, spMCA, spPA, and MCA/PA at different runs suggests that the experimental factors affect response variables in different ways.

The significant *P*-values of spMCA models as well as the insignificant *P*-values of lack-of-fit mean that the models fit well. The *R*^2^ values for the initial and final reduced models for spMCA suggest that the independent variables are responsible for 85.36% and 85.02% of the variation in the response variable (spMCA), as explained by these models, respectively.

The insignificant *P*-value of the MCA/PA model suggests that this ratio cannot be considered as the primary response variable. The *R*^2^ value for the MCA/PA model suggests that the independent variables are responsible for only 57.83% of the variation in the response variable (MCA/PA). Some combinations of enzyme activity values may reflect non-expressive values for this ratio, such as moderate or lower values of MCA combined with too low values of PA, or higher values of MCA combined with higher PA values. On the other hand, the multi-response prediction based on both spMCA and MCA/PA yielded actual response values similar to the predicted values

#### The effect of moisture content

The desired values of response variables were correlated with higher ratios of moisture content. This could be due to the increased nutrient solubility and the higher degree of wheat bran particle swelling which increases its surface area exposed to microbial activity. The optimal moisture content was predicted to be 80%, in contrast to the 50% [26, 29].

#### The effect of pH

The pH level of 3 inhibited enzyme production, and fungal growth was also negligible. Ayhan et al. [10] reported the repression of growth and protease synthesis in the submerged cultures of *Rhizomucor miehei* at similar pH levels. Enzyme activity gradually increased with increasing pH levels reaching the optimal response values at the pH of 6.3, similar to the 6.8 [10].

#### The effect of fermentation time

No enzyme production was observed at the first day of incubation, probably due to insufficient time for spore germination. Both enzyme production and activity were increased after 48 h. Response variables were predicted to be maximized after 81.21 h of incubation, in contrast to the 48 h reported by Araújo et al. [5] and the 110 h reported by Khademi et al. [29]. Proteases are known to be largely produced during the late log phase and the early stationary phase [39]. Escobar and Barnett [22] have reported that *Rhizomucor miehei* cultures reached the stationary phase through 90−120 h. Enzyme yield and activity were decreased after 96 h of incubation, probably due to nutrient depletion, accumulation of organic acids, and lower pH. Similar findings were reported [25, 26, 47].

#### The effect of temperature

No enzyme production was observed at 30 °C, and fungal growth was also negligible. *Rhizomucor miehei* is a thermophilic fungus, and the fungal growth is expected to be enhanced at higher temperature degrees, which reflects on enzyme synthesis. Maximum enzyme activity was observed at 40 °C. Enzyme activity decreased at higher temperature levels, probably due to moisture evaporation from the fermentation medium. Similar findings were reported [26, 29, 46].

#### The effect of casein concentration

The effect of casein was discussed in the preliminary screening step. The effect of casein concentration was not significant against both of spMCA and MCA/PA, probably because of the previous setup near the optimal values. The optimal ratio of casein addition was predicted to be 1.33% w/w, similar to the 1.5% w/w reported by Khademi et al. [29], but in contrast to the 250% w/w reported by Fileto-Pérez et al. [25]. Lower enzyme activity was obtained at lower and higher ratios.

The values of response variables obtained under the optimized conditions are superior to those reported by Preetha and Boopathy [37], Silveira et al. [41], De Lima et al. [18], and Fileto-Pérez et al. [25]. The reported values of response variables signify the high quality of the produced enzyme by the high milk clotting activity and the relatively low proteolytic activity, which is favorable in cheese making.

## Conclusions

The optimal conditions predicted for protease production from *Rhizomucor miehei* were found to be 81.21 h, 41.11°C, 6.31, 80%, and 1.33% for fermentation period, temperature, pH, moisture content, and casein concentration, respectively. The response factor values obtained under the established optimum conditions were 5.11 mg/mL, 2258.13 SU/mL, 441.90 SU/mg, 1.14 PU/mg, and 388.66 for protein content, milk clotting activity, specific clotting activity, specific proteolytic activity, and MCA/PA ratio, respectively; these values were close to the predicted values. The high milk clotting activity and the relatively low proteolytic activity signify higher specificity of the produced enzyme, which is favorable in cheese making. These results reveal the efficiency of the applied statistical approaches in obtaining desired values of response variables and minimizing experimental runs, as well as achieving good predictions for response variables.

We recommend scaling up the production of *Rhizomucor miehei* protease under the aforementioned conditions. Further protease purification and characterization is recommended. We also recommend to compare the characteristics of cheese made with *Rhizomucor miehei* protease and with calf rennet.

## Data Availability

All data generated or analyzed during this study are included in this published article.

## Abbreviations

MCA: Milk clotting activity
PA: Proteolytic activity
MCA/PA: Milk clotting activity to proteolytic activity ratio
spMCA: Specific clotting activity
spPA: Specific proteolytic activity
RSM: Response surface methodology
OFAT: One-factor-at-a-time
CCD: Central composite design
